# 18q loss of heterozygosity in microsatellite stable  colorectal cancer is correlated with CpG island methylator phenotype-negative  (CIMP-0) and inversely with CIMP-low and CIMP-high

**DOI:** 10.1186/1471-2407-7-72

**Published:** 2007-05-02

**Authors:** Shuji Ogino, Takako Kawasaki, Gregory J Kirkner, Mutsuko Ohnishi, Charles S Fuchs

**Affiliations:** 1 Department of Medical Oncology, Dana-Farber Cancer Institute, Boston, MA 02115, USA.; 2 Department of Pathology, Brigham and Women's Hospital, Boston, MA 02115, USA.; 3 Harvard Medical School, Boston, MA 02115, USA.; 4 Department of Medicine, Brigham and Women's Hospital, Boston, MA 02115, USA.

## Abstract

**Background:**

The CpG island methylator phenotype (CIMP) with widespread promoter methylation is a distinct epigenetic phenotype in colorectal cancer, associated with microsatellite instability-high (MSI-high) and *BRAF *mutations. 18q loss of heterozygosity (LOH) commonly present in colorectal cancer with chromosomal instability (CIN) is associated with global hypomethylation in tumor cell. A recent study has shown an inverse correlation between CIN and CIMP (determined by MINTs, p16, p14 and *MLH1 *methylation) in colorectal cancer. However, no study has examined 18q LOH in relation to CIMP-high, CIMP-low (less extensive promoter methylation) and CIMP-0 (CIMP-negative), determined by quantitative DNA methylation analysis.

**Methods:**

Utilizing MethyLight technology (real-time PCR), we quantified DNA methylation in 8 CIMP-specific promoters {*CACNA1G*, *CDKN2A *(p16), *CRABP1, IGF2*, *MLH1, NEUROG1, RUNX3 *and *SOCS1*} in 758 non-MSI-high colorectal cancers obtained from two large prospective cohorts. Using four 18q microsatellite markers (D18S55, D18S56, D18S67 and D18S487) and stringent criteria for 18q LOH, we selected 374 tumors (236 LOH-positive tumors with ≥ 2 markers showing LOH; and 138 LOH-negative tumors with ≥ 3 informative markers and no LOH).

**Results:**

CIMP-0 (0/8 methylated promoters) was significantly more common in 18q LOH-positive tumors (59% = 139/236, p = 0.002) than 18q LOH-negative tumors (44% = 61/138), while CIMP-low/high (1/8–8/8 methylated promoters) was significantly more common (56%) in 18q LOH-negative tumors than 18q LOH-positive tumors (41%). These relations persisted after stratification by sex, location, or the status of MSI, p53 expression (by immunohistochemistry), or *KRAS/BRAF *mutation.

**Conclusion:**

18q LOH is correlated positively with CIMP-0 and inversely with CIMP-low and CIMP-high. Our findings provide supporting evidence for relationship between CIMP-0 and 18q LOH as well as a molecular difference between CIMP-0 and CIMP-low in colorectal cancer.

## Background

Chromosomal instability (CIN) appears to be a distinct phenotype in colorectal cancer, and tumors with CIN show frequent cytogenetic abnormalities and chromosomal gains and losses [1]. CIN is commonly assessed by LOH analyses of microsatellite markers, and markers in the 18q region appear to be generally more sensitive, compared to markers in other chromosomal regions such as 1p, 2p, 3p, 5q, 8p and 17p [2-5]. The presence of 18q LOH has been proposed as a worse prognostic marker for patient survival in colorectal cancer [6-10], and appears to predict resistance to fluorouracil-based chemotherapy [11]. In particular, *SMAD4 *(DPC4), present in 18q21.1 and encoding a downstream signal transducer of TGF-β (transforming growth factor-β), has been implicated as an important tumor suppressor lost by 18q deletion [1,12].

Transcriptional inactivation by cytosine methylation at promoter CpG islands of tumor suppressor genes is thought to be an important mechanism in human carcinogenesis [13]. A number of tumor suppressor genes are silenced by promoter methylation in colorectal cancer [14]. Promoter CpG island methylation has been shown to occur early in colorectal carcinogenesis [15]. A subset of colorectal cancers exhibit promoter methylation in multiple genes, referred to as the CpG island methylator phenotype (CIMP) [14,16]. CIMP-high colorectal tumors appear to have a distinct clinical, pathologic and molecular profile, including associations with female sex, proximal tumor location, poor differentiation, microsatellite instability (MSI) and *BRAF *mutation [17-25]. In addition, we have identified CIMP-low (with less widespread promoter methylation) in colorectal cancer as a phenotype associated with male sex and *KRAS *mutations, different from CIMP-high and CIMP-0 (CIMP-negative) [26]. We have also shown a possible link between CIMP-low, MSI-low, *MGMT *methylation/silencing and *KRAS *mutation, supporting that CIMP-low tumors have unique molecular features different from CIMP-high and CIMP-0 tumors [27].

An association between global hypomethylation and 18q LOH in colorectal cancer has been shown [4]. Genome-wide DNA demethylation has been correlated with chromosomal instability [28-30]. In *Apc*^*Min*/+ ^mice, DNA hypomethylation promotes microadenoma formation through LOH at the *Apc *locus [31]. These studies examined genome-wide DNA methylation, but not DNA methylation in specific promoter CpG islands that is important in silencing of individual tumor suppressor genes. Recently, Goel et al. [32] has shown an inverse correlation between CIN and CIMP in colorectal cancer, using MINT1, MINT2, MINT31, p16, p14 and *MLH1 *methylation markers. However, no study has examined CIN in relation to CIMP-high, CIMP-low and CIMP-0, utilizing quantitative DNA methylation analysis on CIMP-specific promoters recently described by Weisenberger et al. [24].

In this study, utilizing quantitative real-time PCR (MethyLight technology) on recently described CIMP-specific promoters [23,24], and colorectal cancer samples from two large prospective cohort studies, we examined relationship between 18q LOH and CIMP status in non-MSI-H tumors. MethyLight assays can reproducibly distinguish high from low levels of DNA methylation, the latter of which likely have little or no biological significance [33]. We have been able to demonstrate differential relationships of CIMP-low and CIMP-0 with 18q LOH.

## Methods

### Study group

For this study, we utilized the databases of two large prospective cohort studies; the Nurses' Health Study (N = 121,700 women followed since 1976) [34], and the Health Professional Follow-up Study (N = 51,500 men followed since 1986) [35]. Informed consent was obtained from all participants prior to inclusion in the cohorts. A subset of the cohort participants developed colorectal cancers during prospective follow-up. Thus, these colorectal cancers represented population-based, relatively unbiased samples (in contrast to retrospective or single-hospital-based samples). Follow-up of these cohort studies are still ongoing and outcomes data have not been available yet. Previous studies on Nurses' Health Study and Health Professionals Follow-up Study have described baseline characteristics of cohort participants and incident colorectal cancer cases, and confirmed that our colorectal cancer cases were well representative as a population-based sample [34,35]. We collected paraffin-embedded tissue blocks from hospitals where cohort participants with colorectal cancers had undergone resections of primary tumors. We excluded cases if adequate paraffin-embedded tumor tissue was not available at the time of this study. Among our cohort studies, there was no significant difference in demographic features between cases with tissue available and those without available tissue (Chan et al. in press). As a result, a total of 920 colorectal cancer cases were initially included [36]. In only seven other cases, MethyLight reactions failed (failure rate 7/927 = 0.76%). Among the 920 tumors, MSI status could be determined in 889 tumors, and 131 tumors (15%) were MSI-H, thus leaving 758 MSI-L/MSS tumors for further analyses. For this study, we excluded MSI-H tumors because of the presence of widespread unstable microsatellites, and we selected cases only if strictly informative results of 18q microsatellite status were available (see below the method section for MSI and 18q LOH). As a result, 374 colorectal cancer cases (174 from the men's cohort and 200 from the women's cohort) were further studied. Most cases have been previously characterized for status of CIMP, MSI, *KRAS*, and *BRAF *[23,26,36]. However, no tumor has previously been studied for 18q LOH status in relation to MSI and CIMP. Tissue collection and analyses were approved by the Institutional Review Boards.

### Genomic DNA extraction and whole genome amplification

Tumor and normal tissue was dissected from paraffin embedded blocks and genomic DNA was extracted using QIAmp DNA Mini Kit (Qiagen, Valencia, CA USA), as previously described [37]. Normal DNA was derived from a resection margin or a random sampling of normal intestine in a resected specimen. Whole genome amplification of genomic DNA was performed by PCR using random 15-mer primers [37] for subsequent genetic analyses. Previous studies have shown that whole genome amplification does not significantly affect subsequent microsatellite analysis and gene sequencing [37,38].

### Microsatellite instability (MSI) and 18q loss of heterozygosity (LOH)

Methods for MSI analysis were previously described [39]. In addition to D2S123, D5S346, D17S250, BAT25 and BAT26 (the NCI panel), we used BAT40, D18S55, D18S56, D18S67 and D18S487 (i.e., a 10-marker panel); the latter four were located in 18q. "MSI-high (MSI-H)" was defined as instability in 30% or more of the markers, "MSI-low (MSI-L)" as instability in less than 30% of the markers, and "microsatellite stability (MSS)" as no unstable marker.

For LOH analysis, we excluded MSI-H tumors because the presence of unstable microsatellites made LOH analysis difficult. We duplicated PCR reaction and electrophoresis in each sample for D18S55, D18S56, D18S67 and D18S487 to exclude allele dropouts of one of two alleles. LOH at each locus (D18S55, D18S56, D18S67 or D18S487) was defined as 40% or greater reduction of one of two allele peaks in tumor DNA relative to normal DNA [39]. In electropherograms, T1, T2, N1 and N2 were defined as peak heights of allele 1 in tumor DNA, allele 2 in tumor DNA, allele 1 in normal DNA and allele 2 in normal DNA, respectively. LOH at each locus was positive when (T1*N2)/(T2*N1) was ≤ 6/10 in two duplicated runs, or ≥ 10/6 in two duplicated runs.

Overall 18q LOH positivity was strictly defined as the presence of at least two informative markers with LOH, and 18q LOH negativity as the presence of at least three informative markers and no evidence of LOH. These stringent criteria enabled us to select biologically homogenous groups of tumors. When we used less stringent criteria in which 18q LOH positivity was defined as ≥ 1 informative markers with LOH and 18q LOH negativity as ≥ 2 (or ≥ 1) informative markers and no evidence of LOH, the relations between 18q LOH and CIMP became weaker (data not shown).

### Real-time PCR (MethyLight) for quantitative DNA methylation analysis

Sodium bisulfite treatment on genomic DNA was performed as previously described [33]. Real-time PCR to measure DNA methylation (MethyLight) was performed as previously described [40,41]. Utilizing ABI 7300 (Applied Biosystems, Foster City, CA USA) for quantitative real-time PCR, we amplified 8 CIMP-specific promoters, including *CACNA1G*, *CDKN2A *(*p16*), *CRABP1*, *IGF2*, *MLH1*, *NEUROG1, RUNX3 *and *SOCS1 *[23,24]. *COL2A1 *(the collagen 2A1 gene) was used to normalize for the amount of input bisulfite-converted DNA [33,41]. Primers and probes were previously described as follows: *CACNA1G, CRABP1 *and *NEUROG1 *[23,24],*CDKN2A *and *COL2A1 *[41]; *MLH1 *[33]; and *IGF2*, *RUNX3 *and *SOCS1 *[24]. The percentage of methylated reference (PMR, i.e., degree of methylation) at each locus was calculated by dividing the *GENE:COL2A1 *ratio of template amounts in a sample by the *GENE:COL2A1 *ratio of template amounts in *Sss*I-treated human genomic DNA (presumably fully methylated) and multiplying this value by 100 [40]. Methylation positivity in each locus was defined as PMR > 4 (except for *CRABP1 *and *IGF2 *for which positivity was defined as PMR > 6) [23,33,41]. Precision and performance characteristics of bisulfite conversion and subsequent MethyLight assays have been previously evaluated and the assays have been validated [33].

The CpG island methylator phenotype-high (**CIMP-high**) was defined as the presence of ≥ 6/8 methylated promoters, **CIMP-low **as the presence of 1/8 to 5/8 methylated promoters **CIMP-0 **as the absence (0/8) of methylated promoters, based on the previous data that CIMP-high and CIMP-low show high *BRAF *and *KRAS *mutation rates, respectively [26,36].

### Sequencing of *KRAS *and *BRAF*

Methods of *KRAS *and *BRAF *sequencing have been previously described [26,37]. Pyrosequecing was performed using the PSQ96 HS System (Biotage AB and Biosystems, Uppsala, Sweden).

### Immunohistochemistry for p53

Methods of tissue microarray construction [42] and p53 immunohistochemistry have previously been described [43]. p53 positivity was defined as 50% or more of tumor cells with unequivocal strong nuclear staining. All immunohistochemically-stained slides were interpreted by a pathologist (S.O.) blinded from clinical and other laboratory data.

### Statistical analysis

In statistical analysis, chi-square test (or Fisher's exact test when N < 10 in any category) was performed for categorical data, using the SAS program (version 9.1, SAS Institute, Cary, NC). All p values were two-sided, and statistical significance was set at p ≤ 0.05.

## Results

### CIMP-high, CIMP-low and CIMP-0 in non-MSI-H colorectal cancer

**Figure 1:**
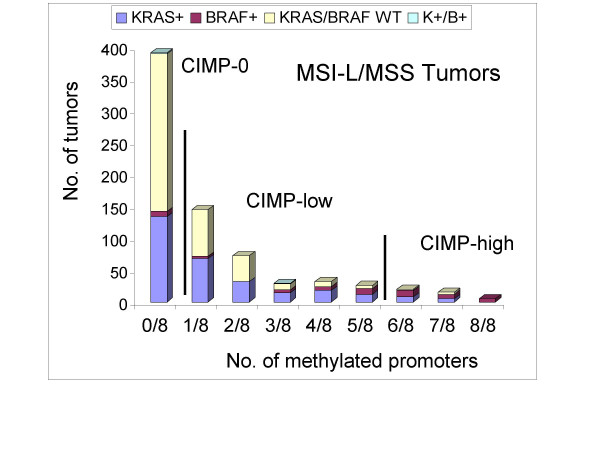
Distribution of MSI-L/MSS colorectal cancers according to the number of methylated promoters. The X and Y axes indicate the number of methylated promoters and the number of tumors, respectively. Based on the *KRAS *and *BRAF *mutation rates, CIMP-high is defined as the presence of ≥ 6/8 methylated promoters. Also note that CIMP-0 tumors with 0/8 methylated promoters are more likely to have both wild-type *KRAS *and *BRAF*, compared to CIMP-low tumors with 1/8 to 5/8 methylated promoters. Abbreviations: BRAF+, *BRAF*-mutated *KRAS *wild-type; KRAS+, *KRAS*-mutated *BRAF *wild-type; K+/B+, *KRAS*-mutated *BRAF*-mutated; KRAS/BRAF WT, both wild-type *KRAS *and *BRAF*; MSI-L, microsatellite instability-low; MSS, microsatellite stable.

We obtained 920 colorectal cancer specimens and quantified DNA methylation in the 8 CIMP-specific gene promoters (*CACNA1G, CDKN2A, CRABP1, IGF2, MLH1, NEUROG1, RUNX3 *and *SOCS1*) by MethyLight technology. All of the 8 loci were selected, based on screening of 195 CpG island loci throughout the human genome [23,24]. The use of the 8 markers as a CIMP diagnostic panel has previously been validated, and all of the 8 markers are sensitive and specific markers for CIMP-high [36]. Among the 920 tumors, MSI status could be determined in 889 tumors, and 758 tumors (85%) were determined as MSI-L/MSS tumors for further analyses. A distribution of MSI-L/MSS tumors according to the number of methylated markers is illustrated in Figure 1, which shows associations between CIMP-high and *BRAF *mutations, between CIMP-low and *KRAS *mutations, and between CIMP-0 with wild-type *BRAF/KRAS*.

### 18q LOH and MSI status

For 18q LOH analysis, we analyzed four 18q microsatellite markers (D18S55, D18S56, D18S67 and D18S487) by duplicated PCR in MSI-L and MSS tumors. To select a biologically homogenous group of tumors (without a considerable number of misclassified tumors), we used stringent criteria for 18q LOH positivity (i.e., the presence of at least two informative markers showing LOH). 18q LOH negativity was also strictly defined as the presence of at least three informative markers and no evidence of LOH. Among the 758 MSI-L/MSS tumors, 374 tumors were classified as either 18q LOH positive (N = 236) or negative (N = 138) by our stringent criteria. 18q LOH was more common in MSI-L tumors (82% = 23/28, p = 0.05) than in MSS tumors (62% = 213/346).

### 18q LOH and methylation in individual CpG islands

We examined the frequency of methylation in each marker according to the 18q LOH status in the MSI-L/MSS tumors (Table 1). The frequency of methylation in each marker was consistently higher in LOH-negative tumors than in LOH-positive tumors. However, no consistent relationship between LOH and methylation in any of the markers was present after tumors were stratified by the CIMP status (data not shown).

### 18q LOH in MSI-L/MSS colorectal cancer is associated positively with CIMP-0 and inversely with CIMP-low/high

**Figure 2:**
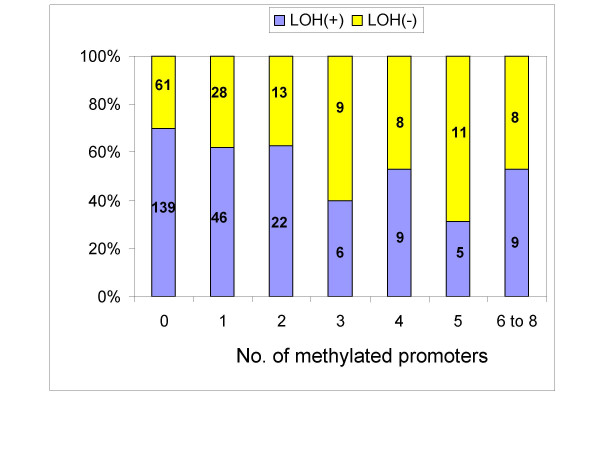
Frequency of 18q LOH in MSI-L/MSS colorectal cancers, according to the number of methylated promoters. CIMP-0 tumors (N = 200) with 0 methylated promoters show the highest frequency of 18q LOH.

We assessed the frequency of 18q LOH in MSI-L/MSS tumors in relation to the number of methylated promoters (Figure 2). The frequency of 18q LOH was highest in CIMP-0 tumors (with 0 methylated promoters), and appeared to decline as the number of methylated promoters increased. To assess relationship between 18q LOH and CIMP, we examined the frequencies of CIMP-0 in 18q LOH-positive tumors and 18q LOH-negative tumors (Table 2). Among the 374 MSI-L/MSS tumors, CIMP-0 was significantly more common in 18q LOH-positive tumors (59%, p = 0.002) than in 18q LOH-negative tumors (44%). Even after tumors were stratified by MSI status, 18q LOH-positive tumors consistently showed a higher frequency of CIMP-0 than 18q LOH-negative tumors.

### Relationship between 18q LOH and CIMP-0 persists after various stratifications

To examine whether the relationship between 18q LOH and CIMP status was affected by clinical or other molecular variables, we stratified tumors by sex, tumor location, p53 status or *KRAS/BRAF *status, and examined the frequencies of CIMP-0 in 18q LOH positive or negative tumors (Table 3). Interestingly, 18q LOH positive tumors consistently showed a higher frequency of CIMP-0 and a lower frequency of CIMP-low/high than 18q LOH negative tumors in all of the 9 categories (male, female, right colon, left colon, etc.; 11 categories including MSI-L and MSS, p = 0.0005), further supporting the positive correlation between 18q LOH and CIMP-0.

### Frequency of 18q LOH in various MSI/CIMP subtypes of colorectal cancer

**Figure 3:**
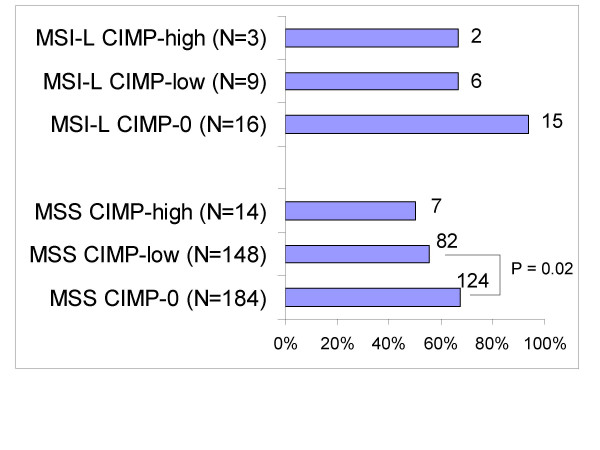
Frequency of 18q LOH in MSI/CIMP subtypes of colorectal cancer. CIMP-0 tumors show higher frequencies of 18q LOH than CIMP-low and CIMP-high tumors within the MSI-L and MSS subgroups. Abbreviations: CIMP, CpG island methylator phenotype; LOH, loss of heterozygosity; MSI-L, microsatellite instability-low; MSS, microsatellite stable.

A molecular classification of colorectal cancer based on MSI and CIMP status is increasingly important, because MSI and CIMP status reflect global genomic and epigenomic aberrations, respectively. We evaluated the frequency of 18q LOH in relation to combined MSI and CIMP status (Figure 3). Either among MSI-L or MSS tumors, CIMP-0 tumors showed a higher frequency of 18q LOH than CIMP-low and CIMP-high tumors.

## Discussion

We conducted this study to examine 18q loss of heterozygosity (LOH) in relation to the CpG island methylator phenotype (CIMP) in colorectal cancer. Discovering molecular correlates is important in cancer research, because it may: (1) provide clues to pathogenesis; (2) propose or support the existence of a new molecular subtype; (3) alert investigators to be aware of potential confounding in association studies; and (4) suggest surrogate markers in clinical or research settings. In the current study, our strict criteria for 18q LOH could select biologically more homogenous groups of samples, enabling us to identify interesting molecular correlates that might otherwise have been obscured or missed. The use of quantitative DNA methylation assays (MethyLight) on the 8 carefully-selected CIMP-specific promoters [23,24] as well as population-based samples of colorectal cancer from two large prospective cohort studies [34,35] has enabled us to accurately estimate the frequency of colorectal cancers with a specific molecular feature (e.g., CIMP-0).

We have demonstrated that 18q LOH is correlated positively with CIMP-0 (with 0/8 methylated promoter) and inversely with CIMP-low (1/8 to 5/8 methylated promoters). Our data are somewhat in agreement with the data by Goel et al. [32], who discovered an inverse correlation between LOH and CIMP. However, there are differences in methods and results between our study and Goel et al's study [32] that warrant discussion. Although more LOH markers were analyzed, Goel et al. [32] used non-quantitative methylation-specific PCR (MSP) assays on MINT1, MINT2, MINT31, p16 (CDKN2A), p14 (ARF), *MLH1 *(and additional non-CIMP related markers). The authors found only several CIMP-0 tumors (with no methylated promoter), which were too small in number to conclude a significant difference between CIMP-0 and CIMP-low. In the current study, we could demonstrate the significant difference between CIMP-low and CIMP-0 in MSI-L/MSS tumors. There might be several reasons for the discrepancies, including differences in the sample sizes, the methylation assays (MSP vs. MethyLight) and the methylation markers (MINTs vs. CIMP-high-specific markers). The use of MSP could overestimate the frequency of methylation positivity in any marker. We have shown that low-level CpG island methylation below the threshold of gene silencing should be reproducibly distinguished from high-level methylation by quantitative DNA methylation analysis [33]. The methylation markers MINT1, MINT2 and MINT31 were originally described as the markers for CIMP [16]. A recent extensive study by Weisenberger et al. [24] has shown that MINT1, MINT2 and MINT31 are not as specific for *BRAF *mutations as our markers, *CACNA1G, CDKN2A (p16), IGF2, MLH1, NEUROG1, RUNX3 *and *SOCS1*. By the use of the latter markers, we could separate CIMP-high tumors (with frequent *BRAF *mutations) from CIMP-low tumors (with frequent *KRAS *mutations) (Figure 1).

Our data provide supporting evidence for a molecular difference between CIMP-low and CIMP-0 (CIMP-negative) in colorectal cancer. We have previously shown that CIMP-low tumors are associated with male sex and *KRAS *mutation, whereas CIMP-high tumors are associated with female sex and *BRAF *mutation, and CIMP-0 tumors are associated with wild-type *KRAS/BRAF *oncogenes and exhibits no sex predilection [26]. We have recently shown that the relationship between MSI-low and *MGMT *methylation/silencing is present only in CIMP-low tumors, but not in CIMP-high or CIMP-0 tumors, supporting unique molecular features of CIMP-low different from CIMP-high and CIMP-0 [27]. A significant difference in the *BRAF *mutation frequencies between CIMP-high and CIMP-low tumors has repeatedly been shown by other investigators [20-22,24]; however, a difference between CIMP-low and CIMP-0 has not been widely investigated. We admit that the difference between CIMP-low and CIMP-0 is not as clear-cut as that between CIMP-high and CIMP-low. Thus, additional studies are necessary to identify a panel of markers specific for CIMP-low to be clearly distinguished from CIMP-0.

The relationship between global DNA methylation level (but not specific CpG island methylation) and chromosomal aberrations has been studied. In a study using *Apc*^*Min*/+ ^mice, Yamada et al. [31] have shown that hypomorphic alleles of the *Dnmt1 *gene cause DNA hypomethylation in intestinal cells leading to increased microadenoma formation through LOH at the *Apc *locus, suggesting a pathogenetic link between DNA hypomethylation and LOH. Genome-wide DNA demethylation has been correlated with chromosomal instability and genomic copy number changes [28,29]. Genetic disruption of DNA methyltransferase or treatment with 5-aza-deoxycytidine results in chromosomal translocations or rearrangements [30,44]. Matsuzaki et al. [4] have examined global DNA methylation levels in colorectal cancer by examining long interspersed nucleotide elements (LINE), and correlated methylation data with the status of MSI and LOH at 5q, 8p, 17p and 18q. The authors showed that the presence of LOH was correlated with global hypomethylation in colorectal cancer, and that the association between 18q LOH and global hypomethylation was particularly strong compared to the associations between the other LOH markers and global hypomethylation [4]. Thus, 18q LOH appeared to be a good predictor for global hypomethylation. Interestingly, our current study has shown that 18q LOH is correlated with CIMP-0 (i.e., no methylation in the 8 CIMP-specific CpG islands). Together with the data by Matsuzaki et al. [4], there may be a possibility that a global methylation level may positively correlate with DNA hypermethylation at specific CpG islands (i.e., CIMP status). Matsuzaki et al. [4] have also shown that MSI-H tumors exhibit a higher global methylation level than MSS tumors though they have not examined *MLH1 *promoter methylation. These data also support the positive correlation between a global methylation level and specific promoter CpG island methylation, because *MLH1 *promoter methylation is the most common cause for sporadic MSI-H tumors and also very specific marker for CIMP-high [23].

## Conclusion

We have demonstrated that 18q LOH in non-MSI-H colorectal cancer is correlated positively with CIMP-0, and inversely with CIMP-low and CIMP-high. Our data provide additional evidence to support a biological difference between CIMP-0 and CIMP-low in colorectal cancer. Our findings of the link between LOH and CIMP-0 indicate that potential confounding effects of CIMP status need to be considered when one examines relationship between allelic imbalance (or chromosomal instability) and patient outcomes or any other clinical or molecular variables. Additional studies will shed lights on the possible pathogenetic link between CIMP-0 and chromosomal instability in colorectal cancer.

## Abbreviations and HUGO Gene Nomenclature Committee (HGNC)-approved official gene symbols used

CACNA1G, calcium channel, voltage-dependent, T type alpha-1G subunit

CDKN2A, cyclin-dependent kinase inhibitor 2A (p16, also known as INK4A)

CIMP, CpG island methylator phenotype

CIN, chromosomal instability

CRABP1, cellular retinoic acid binding protein 1

IGF2, insulin-like growth factor 2

LOH, loss of heterozygosity

MSI, microsatellite instability

MSI-H, microsatellite instability-high

MSI-L, microsatellite instability-low

MSS, microsatellite stable

NEUROG1, neurogenin 1

PMR, percentage of methylated reference (degree of DNA methylation)

RUNX3, runt-related transcription factor 3

SOCS1, suppressor of cytokine signaling 1

## Competing interests

The author(s) declare that they have no competing interests.

## Authors' contributions

SO conceived the study, designed assays, analyzed and interpreted the data, and drafted the manuscript. TK designed and performed the assays, and analyzed and interpreted the data. GJK coordinated the study, and analyzed and interpreted the data. CSF coordinated the study, discussed the study design and data, and drafted the manuscript.  MO provided statistical support and edited to revise the  manuscript.

## Pre-publication history

The pre-publication history for this paper can be accessed here:



## Figures and Tables

**Table 1:** Frequency of methylation in each marker according to 18q LOH status in MSI-L/MSS colorectal cancers

18q LOH status	Total N	No. of tumors with methylation in:							
		*CACNA1G*	*CDKN2A (p16)*	*CRABP1*	*IGF2*	*MLH1*	*NEUROG1*	*RUNX3*	*SOCS1*
LOH(+)	236	23 (9.7%)	55 (23%)	44 (19%)	28 (12%)	4 (1.7%)	44 (19%)	15 (6.4%)	15 (6.4%)
LOH(-)	138	26 (19%)	38 (28%)	47 (34%)	30 (22%)	3 (2.2%)	41 (30%)	23 (17%)	14 (10%)

Abbreviations: LOH, loss of heterozygosity; MSI-L, microsatellite instability-low; MSS, microsatellite stable.

**Table 2:** Frequencies of CIMP-0 and CIMP-low/high in MSI-L/MSS colorectal cancer with or without 18q LOH

	18q LOH status	Total N	CIMP-0 (0/8)^a^	CIMP-low/high (1/8 – 8/8)^a^	P value	CIMP-low (1/8 – 5/8)^a^	CIMP-high (6/8 – 8/8)^a^
All cases		920	431 (47%)	489 (53%)		353 (38%)	136 (15%)
MSI-L/MSS	(+)	236	139 (59%)	97 (41%)	0.002	88 (37%)	9 (3.8%)
	(-)	138	61 (44%)	77 (56%)		69 (50%)	8 (5.8%)
MSI-L	(+)	23	15 (65%)	8 (35%)		6 (26%)	2 (8.7%)
	(-)	5	1 (20%)	4 (80%)		3 (60%)	1 (20%)
MSS	(+)	213	124 (58%)	89 (42%)	0.01	82 (38%)	7 (3.3%)
	(-)	133	60 (45%)	73 (55%)		66 (50%)	7 (5.3%)

^a ^Fraction of the number of methylated promoters.

CIMP, CpG island methylator phenotype; LOH, loss of heterozygosity; MSI-L, microsatellite instability-low; MSS, microsatellite stable.

**Table 3:** Frequency of CIMP in MSI-L/MSS tumors with or without 18q LOH after various stratifications

	18q LOH status	Total N	CIMP-0 (0/8)^a^	CIMP-low/high (1/8 – 8/8)^a^	P value	CIMP-low (1/8 – 5/8)	CIMP-high (6/8 – 8/8)
Men	(+)	104	59 (57%)	45 (43%)	0.02	41 (39%)	4 (3.8%)
	(-)	70	28 (40%)	42 (60%)		39 (56%)	3 (4.3%)
Women	(+)	132	80 (61%)	52 (39%)		47 (36%)	5 (3.8%)
	(-)	68	33 (49%)	35 (51%)		30 (44%)	5 (7.4%)
Right colon	(+)	45	19 (42%)	26 (58%)		23 (51%)	3 (6.7%)
	(-)	37	12 (32%)	25 (68%)		21 (57%)	4 (11%)
Left colon	(+)	87	54 (62%)	33 (38%)		32 (36%)	1 (1.9%)
	(-)	34	16 (47%)	18 (53%)		18 (53%)	0
p53(+)^	(+)	122	73 (60%)	49 (40%)		48 (39%)	1 (0.8%)
	(-)	43	19 (44%)	24 (56%)		22 (51%)	2 (4.7%)
p53(-)^	(+)	113	66 (58%)	47 (42%)	0.03	39 (35%)	8 (7.1%)
	(-)	90	39 (43%)	51 (57%)		45 (50%)	6 (6.7%)
*KRAS*(-) *BRAF*(-)	(+)	142	93 (65%)	49 (35%)		47 (33%)	2 (1.4%)
	(-)	51	30 (59%)	21 (41%)		21 (41%)	0
*KRAS*(+) *BRAF*(-)	(+)	71	39 (55%)	32 (45%)		31 (44%)	1 (1.4%)
	(-)	72	30 (42%)	42 (58%)		39 (54%)	3 (4.2%)
*KRAS*(-) *BRAF*(+)	(+)	17	3 (18%)	14 (82%)		8 (47%)	6 (35%)
	(-)	12	0	12 (100%)		7 (58%)	5 (42%)

The frequency of CIMP-0 is consistently higher in 18q LOH(+) tumors than in 18q LOH(-) tumors among the 9 different categories (men, women, right colon, etc.; 11 categories including MSI-L and MSS, p = 0.0005).

Footnote: ^a ^Fraction of the number of methylated promoters. ^ p53 status was assessed by immunohistochemistry.

CIMP, CpG island methylator phenotype; LOH, loss of heterozygosity; MSI-L, microsatellite instability-low; MSS, microsatellite stable.
